# PET/MR in children. Initial clinical experience in paediatric oncology using an integrated PET/MR scanner

**DOI:** 10.1007/s00247-012-2570-4

**Published:** 2013-01-11

**Authors:** Franz Wolfgang Hirsch, Bernhard Sattler, Ina Sorge, Lars Kurch, Adrian Viehweger, Lutz Ritter, Peter Werner, Thies Jochimsen, Henryk Barthel, Uta Bierbach, Holger Till, Osama Sabri, Regine Kluge

**Affiliations:** 1 Department of Paediatric Radiology, University of Leipzig, Liebigstr. 20a, 04103 Leipzig, Germany; 2 Department of Nuclear Medicine, University of Leipzig, Liebigstr. 18, 04103 Leipzig, Germany; 3 Department of Paediatric Oncology, University of Leipzig, Liebigstr. 20a, 04103 Leipzig, Germany; 4 Department of Paediatric Surgery, University of Leipzig, Liebigstr. 20a, 04103 Leipzig, Germany

**Keywords:** Children, PET/MR, MR/PET, Oncology, Hybrid imaging

## Abstract

Use of PET/MR in children has not previously been reported, to the best of our knowledge. Children with systemic malignancies may benefit from the reduced radiation exposure offered by PET/MR. We report our initial experience with PET/MR hybrid imaging and our current established sequence protocol after 21 PET/MR studies in 15 children with multifocal malignant diseases. The effective dose of a PET/MR scan was only about 20% that of the equivalent PET/CT examination. Simultaneous acquisition of PET and MR data combines the advantages of the two previously separate modalities. Furthermore, the technique also enables whole-body diffusion-weighted imaging (DWI) and statements to be made about the biological cellularity and nuclear/cytoplasmic ratio of tumours. Combined PET/MR saves time and resources. One disadvantage of PET/MR is that in order to have an effect, a significantly longer examination time is needed than with PET/CT. In our initial experience, PET/MR has turned out to be an unexpectedly stable and reliable hybrid imaging modality, which generates a complementary diagnostic study of great additional value.

## Introduction

Whole-body PET/MR scanners are currently available from two companies and have been in clinical use since 2010: the GEMINI TF PET/MR imaging system (Philips Medical Systems, Eindhoven, The Netherlands) and the Biograph mMR (Siemens, Erlangen, Germany). The technical solution and philosophy between the two scanners are different. With the Gemini TF PET/MR scanner, the PET and MR scans are carried out in quick succession in the same room and on the same moving table, but on two different devices. The mMR scanner is the first available fully integrated whole-body PET/MR system.

It should be emphasized that up to September 2011 both devices were used exclusively for research, and were optimized in research units with the help of the developers and manufacturers, and with the guidance of physicians with regard to their clinical needs. The first regular CE-certified device for clinical use was installed in 2011 at the University Hospital of Leipzig, Germany [1, 2]. Under the auspices of the Department of Nuclear Medicine, the various partners are in the process of carrying out both clinical research projects and examinations.

PET/CT imaging is widely used even in paediatric oncology. Switching from PET/CT to PET/MR would reduce the radiation exposure caused by the CT part of the examination. This is one of the most important aspects for those who have supported the establishment and application of PET/MR in children [3, 4]. Since November 2011, all examinations conducted in children with oncological problems requiring whole-body hybrid imaging in our institution have been performed exclusively using PET/MR (21 examinations). To date, no imaging protocols or information about the feasibility of whole-body PET/MR in children have been published. The aim of this report was to share our initial experience with this innovative technique.

## Imaging technique

The basic idea of combining all the advantages of PET with those of MR in only one examination has been realized by two manufacturers using two very different solutions. The older configuration (Philips) connects two devices consecutively. The patient is examined on the same examination table by time-delayed MR and PET, and the analysed data are then merged. The advantage of using this method is that any interference or crosstalk between PET and MR is easier to avoid. However, with this tandem configuration, simultaneous data acquisition from both modalities is not possible. The newer alternative setup is an integrated whole-body PET/MR system (Siemens). The advantage of this technical solution is that the acquisition of PET and MR data synchronously allows true simultaneous registration of morphological and metabolic processes that follow the same timeline, and results in a significantly improved coregistration of the two modalities [5]. In addition, the simultaneous acquisition of data generates results in a shorter examination time and therefore results in higher patient throughput, as shown by von Schulthess and Burger [6]. Shorter examination times for evaluating treatment response in children are, in particular, highly relevant in clinical practice.

The setup of the fully integrated whole-body PET/MR system we use for our investigations corresponds to the outer structure of the equivalent commercial 3-T unit. The PET detector ring is integrated between the patient-oriented radiofrequency body coil and the gradient coil. Other coils, e.g. the main magnetic coils, are arranged as in other MR machines in the cooled area of the outer mMR [7]. To ensure compatibility between the two devices, and to create as little interference or crosstalk as possible between the MR and PET units, the patient-oriented RF coils, the RF body coil and the PET detectors were designed from scratch. In practice, in comparison to a comparable conventional 3-T system (Trio; Siemens, Germany), any alteration of the MR signal is not noticeable. Identical sequence parameters can be selected for both units. The sequences and parameters are reproducible from the 3-T equipment and are, therefore, very easy to transfer. As a result, the established standard diagnostic MR protocol can, in principle, be carried out completely on the mMR system. Even in young children, 3-T MR devices, compared to 1.5-T devices, have been very successful. The smaller body diameter in children in the abdominal and thoracic areas means that the typical 3-T signal loss artefacts seen in adults are less problematic, and the high field strength leads to less time required for the investigation.

**Fig. 1:**
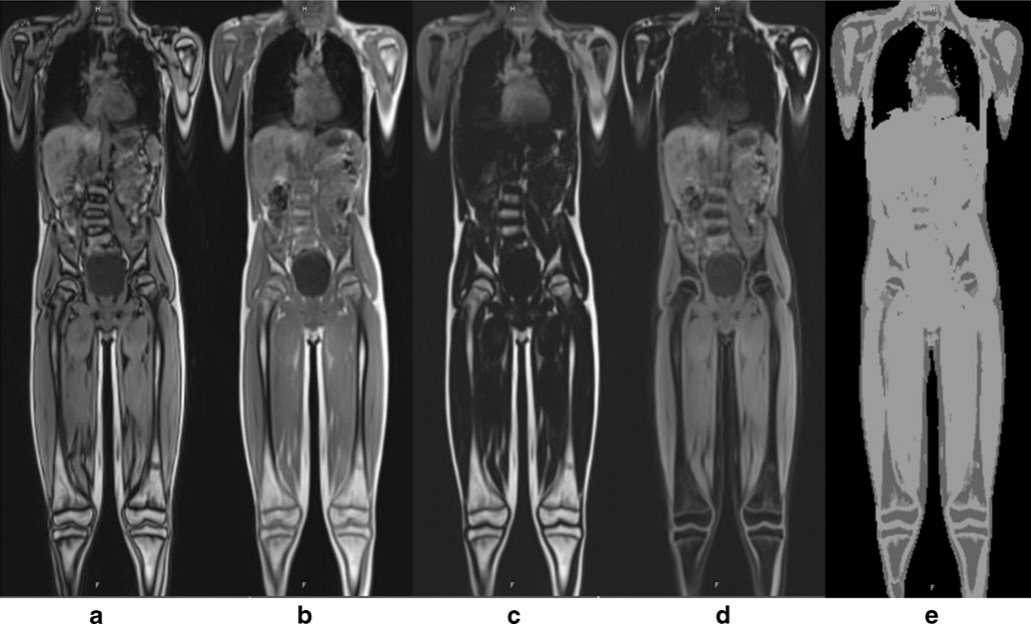
Two-point volume interpolated breathhold examination (VIBE) Dixon sequence for determination/segmentation of fat, lung tissue, air and water (soft tissue) and assignment of a map of linear attenuation coefficients (μ-map). All four components of the sequence are required for calculating an attenuation correction pattern (μ-map) for the PET data: in-phase and opposed-phase T1 images (**a, b**), and fat image and water image (**c, d**). From these images, the μ-map that is utilized for attenuation correction of the PET data is generated/segmented (**e**). For the subsequent PET/MR fusion, only the attenuation-corrected PET data are used

For PET data acquisition, newly developed detectors are used (avalanche photodiodes, APD) whose performance does not suffer from the influence of the magnetic field. These diodes have a size of 5 × 5 mm. Typically, a whole-body measurement using PET/MR is performed in children in seven sections of 30 cm each. With overlapping areas, this results in a maximal total length of about 160 cm. The MR data acquisition lasts 5.5 min per section and consists of two T2 turbo inversion recovery magnitude (TIRM) sequences—transverse and coronal—and a transverse diffusion-weighted sequence. The PET acquisition time is adapted to the measurement time of the MR examination. This does not lead to prolongation of data acquisition. An additional measurement time must be allowed for the measurement and calculation of the attenuation correction for PET and for the “shimming” of the magnetic field [8]. The necessary PET attenuation correction is carried out per section with a MR Dixon sequence consisting of four individual measurements (water image, fat image, and in-phase and opposed-phase T1 images) from which a μ-map is generated (Fig. 1) [9, 10]. This μ-map is then utilized for attenuation correction of the PET data.

In PET/MR, the radiation exposure is from the PET component and is 3–4 mSv in 3-D mode [11, 12]. In contrast, in diagnostic PET/CT, the typical effective dose for a child is about 25 mSv (range: 6.2 to 60.7 mSv). The additional dose fraction with whole-body CT is highly dependent on the selected imaging protocol and thus exposure parameters, and is on average 20 mSv (range: 2.7 to 54.2 mSv). Therefore, it is likely that the radiation exposure from a PET/MR scan is 80% less than that from a PET/CT scan. It is also important to consider that children with oncological diseases often require multiple hybrid imaging studies (mean: 3.2 studies, range: 1–14) [13].

### Imaging protocols

Due to the novelty of the integrated PET/MR system, no recommendation for an imaging protocol in children has yet been published. We therefore developed a child-specific sequence protocol based on our experience with whole-body MR in children at 1.5 and 3 T and paediatric PET/CT [11, 14]. In establishing our investigation strategy, we pursued three premises that appeared to us to be absolutely necessary for a PET/MR diagnostic procedure:

1. Hybrid PET/MR imaging should not lead to diagnostic degradation in either the MR or PET imaging. The anatomical information, which is of particular importance for the paediatric surgeon, must be maintained without any modifications or restrictions.
2. The time for a whole-body examination should be acceptable to the child (45 to 60 min).
3. PET and MR should not just be performed for additive benefit, but should also offer a new, complementary diagnostic quality and clear diagnostic profit.

To achieve these partly contradictory objectives, a compromise was necessary that had to be found in collaboration with clinical colleagues (paediatric surgeons, paediatricians), nuclear medicine physicians and paediatric radiologists. In particular, a numerical and temporal limitation of the MR sequences was necessary, but without reducing the diagnostic safety for the child [15]. The increased field strength of 3 T was used to reduce the time required for the sequences. A simultaneous gain in image quality, compared to 1.5 T, is currently not possible or aimed for.

Our PET/MR protocol is performed as a complementary combination of whole-body imaging to determine the spread of the cancer and includes additional regional MR imaging to give more detailed visualization for surgical procedures [16]. Supplementing the whole-body examination, these additional sequences allow a more dedicated local investigation of the tumour when necessary. The anatomical coverage of the whole-body MR scan is variable; the maximum measurable length in the mMR system is 160 cm. If required, the lower legs and feet can be investigated separately in a second step after repositioning.

Our current sequence concept includes the following sequences:

1. Water-sensitive fast inversion recovery sequence (e.g. TIRM): coronal, 5-mm slice thickness; transverse, 4.5-mm slice thickness (for details, see Table 1). The thoracic and upper abdominal sections are acquired with respiratory triggering using a breathing belt.
2. Whole-body diffusion-weighted imaging at a slice thickness of 4.5 mm and for calculation of the ADC (B-value 800 s/mm^2^) [17].
3. Further, dedicated sequences are performed depending on the problem; the following supplementary sequences are typically used depending on the region:
  T1-weighted spin echo with fat saturation after contrast agent administration as a transverse whole-body examination
  Head: T2-weighted FLAIR, transverse
  Chest: T2-weighted turbo spin echo, transverse, respiratory-triggered
  Abdomen: volume interpolated breathhold examination (VIBE) gradient echo sequence (e.g. VIBE dynamic, three phases after contrast agent administration) and a 2-D T1-weighted fast spoiled gradient echo sequence (e.g. FLASH 2-D, respiratory-triggered)
  Pelvis: T1-weighted spin echo with fat saturation, transverse

**Table 1:** Sequence parameters of the whole-body MR protocol

Parameter	T2-W TIRM, coronal	T2-W TIRM, transverse
Distance factor (%)	10	0
Image orientation	Coronal	Transverse
Phase-encoding direction	Feet → head	Anterior → posterior
Phase oversampling	50	10
Inversion time (ms)	220	220
Field of view	500 mm	Patient-adapted
Phase field of view (%)	50	70
Slice thickness (mm)	5	4.5
Repetition time (ms)	2,090	2,000
Echo time (ms)	47	48
Number of averages	1	1
Flip angle (°)	150	133
Base resolution	320	384
Phase resolution (%)	75	75
Parallel acquisition technique	GRAPPA 2	GRAPPA 2
Turbo factor	16	16
Voxel size	2.1 × 1.6 × 5	1.7 × 1.3 × 4.5
Time per stack (min)	1:08	1:32
Stacks/bed position	7	7
Data acquisition time with respiratory triggering (min, approx)	15	25

Concerning our PET/MR imaging in children, with regard to the MR sequences, the following experiences are worth mentioning:

1. T2-weighted TIRM sequences are superior to spectral fat saturation, as the fat saturation and the shim process are more stable.
2. T1-weighted gradient echo sequences are used in the abdominal region due to a preferred shorter acquisition time, sometimes even only as a complementary T1-weighted VIBE sequence.
3. The average number of acquisitions can be reduced because of the high field strength; owing to the high field strength parallel imaging in most locations is possible and necessary.
4. All sequences in the thoracic region and upper abdomen were performed exclusively with respiratory triggering. In our experience, a respiratory belt is much more stable than navigator sequences in children, since the mean position of the diaphragm changes frequently during the lengthy examination. The repetition time for T2-weighted sequences should be minimized (typically 2,000 ms), which on the one hand leads to a significantly shorter examination time and on the other hand is more suitable for the faster breathing cycle of young children at 25–30 breaths per minute [18].

Patient preparation, FDG dose and the PET acquisition protocol follow the guidelines of the European and German societies of nuclear medicine for FDG PET examinations in children [11, 19]. Imaging started 70 ±15 min after intravenous FDG administration. Doses were in the range 42–371 MBq depending on body weight. Images covered the patient from the head to the middle of the upper legs and generally involved seven bed positions.

## Preliminary findings

Using the Siemens mMR scanner, a total of 21 FDG PET/MR scans were carried out in 15 children (12 boys, 3 girls) between November 2011 and July 2012. The children’s ages ranged from 1 year 11 months to 16 years 8 months (mean age: 10 years 7 months). Oncological diseases included non-Hodgkin lymphoma (*n* = 4), Hodgkin lymphoma (*n* = 3), neuroblastoma (*n* = 2), primitive neuroectodermal tumour (*n* = 1), Ewing sarcoma (*n* = 1), soft tissue sarcoma (*n* = 2), acute myeloid leukaemia with extramedullary manifestation (*n* = 1) and metastasized germ cell tumour of the testis (*n* = 1). Ten of the children were investigated before the start of systemic treatment for initial staging. Responses to treatment were assessed in 11 PET/MR investigations.

The technical success rate was 100%. This relates to the successful and simultaneous acquisition of MR and PET data. Clear advantages of PET/MR over PET/CT and additional MRI are the lower radiation dose and the reduced number of investigations in the children, decreasing the psychological stress and the length of the diagnostic period before the start of treatment. Any additional diagnostic impacts of synchronous PET/MR need to be clarified in future studies. However, in comparison to the use of PET/CT alone, PET/MR has advantages, especially in the description of the anatomical details of primary tumours due to the higher soft tissue contrast of MR imaging.

In the first months of clinical use, PET/MR led to considerable simplification of the practical course of action in children with multifocal oncological diseases. The diagnostic process generally required only one examination (maximum two), which was significantly more acceptable to the children than previous sequential diagnostic procedures. Previous sequential procedures included a local MR scan, a PET or a PET/CT scan, normally a whole-body MR scan and sometimes a thoracic CT scan. This improvement in workflow led to a real one-stop-shop diagnostic procedure that replaced the previous multiple examinations. PET/MR produces only partially redundant, but more commonly additive, information. These results were elucidated in a joint findings discussion between radiologists and nuclear physicians, and then communicated in a joint findings report to clinical colleagues. A particularly positive visual impact was observed when the pathological findings were demonstrated to treating physicians on presentation of the fused images combining the hyperintensity shown by MR and the colour-coded metabolic hyperactivity shown by PET.

### Lymphomas in children

Six children have been investigated for initial staging of lymphoma (including Hodgkin lymphoma, Burkitt lymphoma and T-cell lymphoma; Table 2).

**Table 2:** Number of involved sites and tumour stage (Ann Arbor-classification) diagnosed with the PET component, the MRI component and by combined PET/MR

Patient	Histology	Involved sites		Stage		
		PET	MRI	PET	MRI	Final
1	Classic Hodgkin lymphoma	7	5	II	II	II
2	Classic Hodgkin lymphoma	7	6	II	II	II
3	Lymphocyte-predominant Hodgkin lymphoma	2	1	II	I	II
4	Burkitt lymphoma	5	3	II	III	II
5	T-cell non-Hodgkin lymphoma	5	9	IV	IV	IV
6	T-cell non-Hodgkin lymphoma	10	8	IV	IV	IV

PET and MR components concordantly detected 25 tumour-involved areas. PET detected 10 more metabolically active lymph node areas, and MRI showed three additionally involved organs in one child (Fig. 4) and two additional lymph node sites suspicious for tumour. In two of the six children, the tumour restaged by joint interpretation of PET/MR. One child with lymphocyte-predominant Hodgkin lymphoma had histologically confirmed involvement of the left inguinal nodes. However, PET also showed enhanced FDG uptake suspicious for tumour in the right inguinal nodes of borderline size, thus upstaging the child from stage I to stage II. In a child with Burkitt lymphoma, MR detected a 2.3-mm inguinal lymph node. Due to normal glucose uptake on PET, this node was considered not involved and the child was assigned to stage II.

**Fig. 2:**
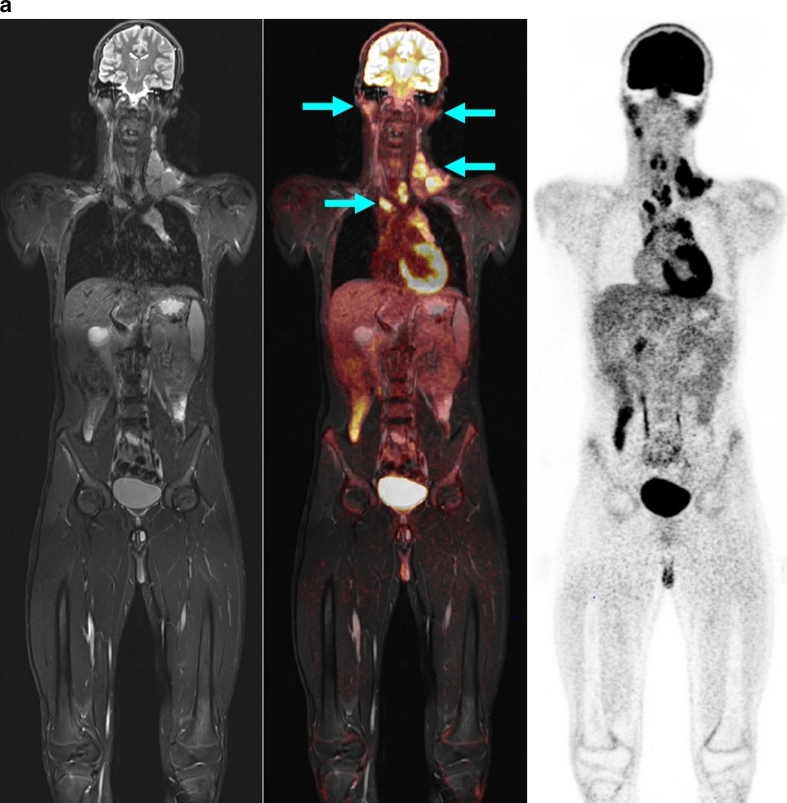

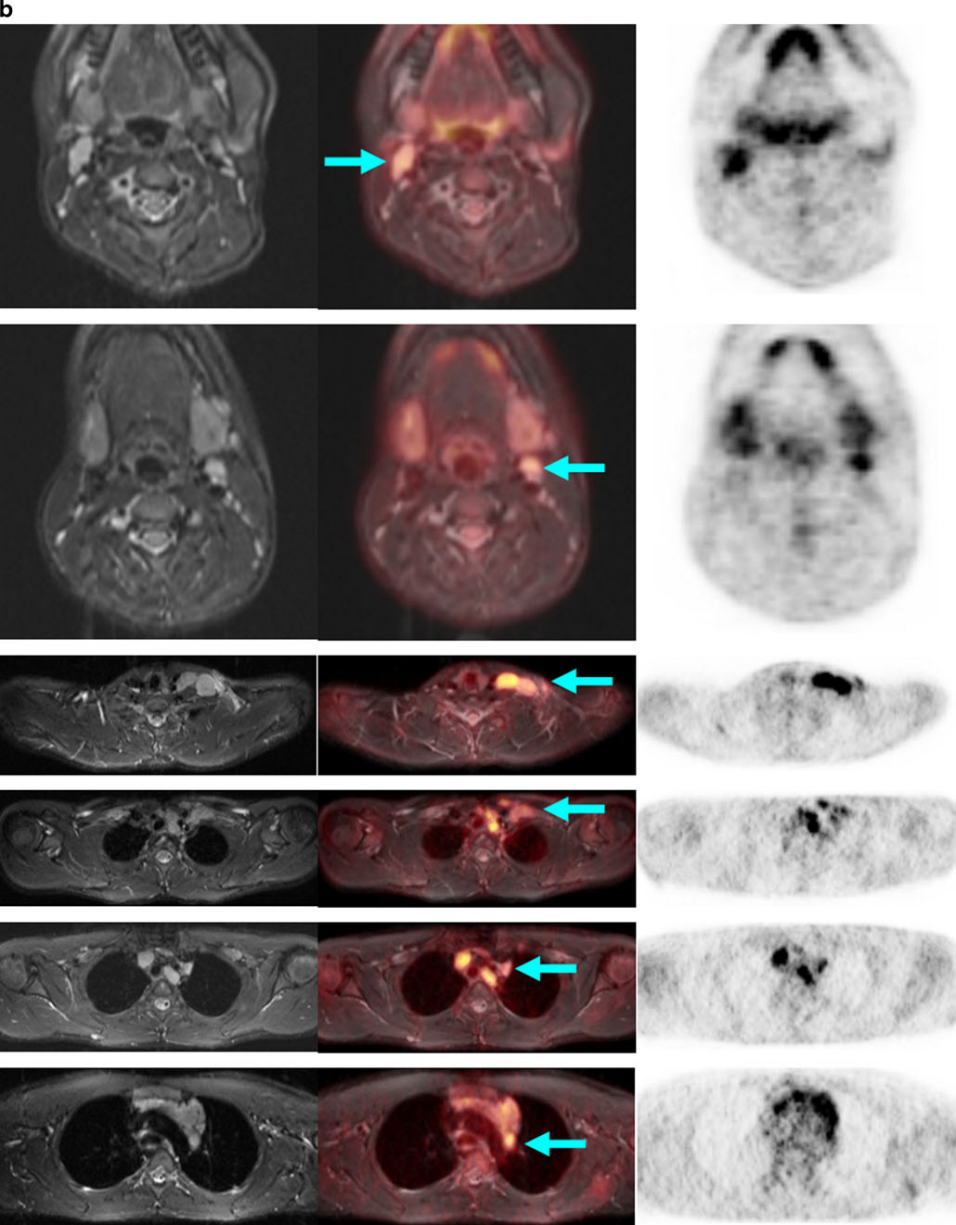
A 13-year-old boy with Hodgkin disease stage II. **a** Involvement of both sides of the submandibular, cervical, and upper and middle mediastinum (*arrows*). **b** Involvement of the submandibular lymph nodes (*arrows*) was not positively identified on MR criteria (size), but the PET image shows significantly enhanced FDG uptake. *Arrows* indicate positive PET/MR findings

**Fig. 3:**
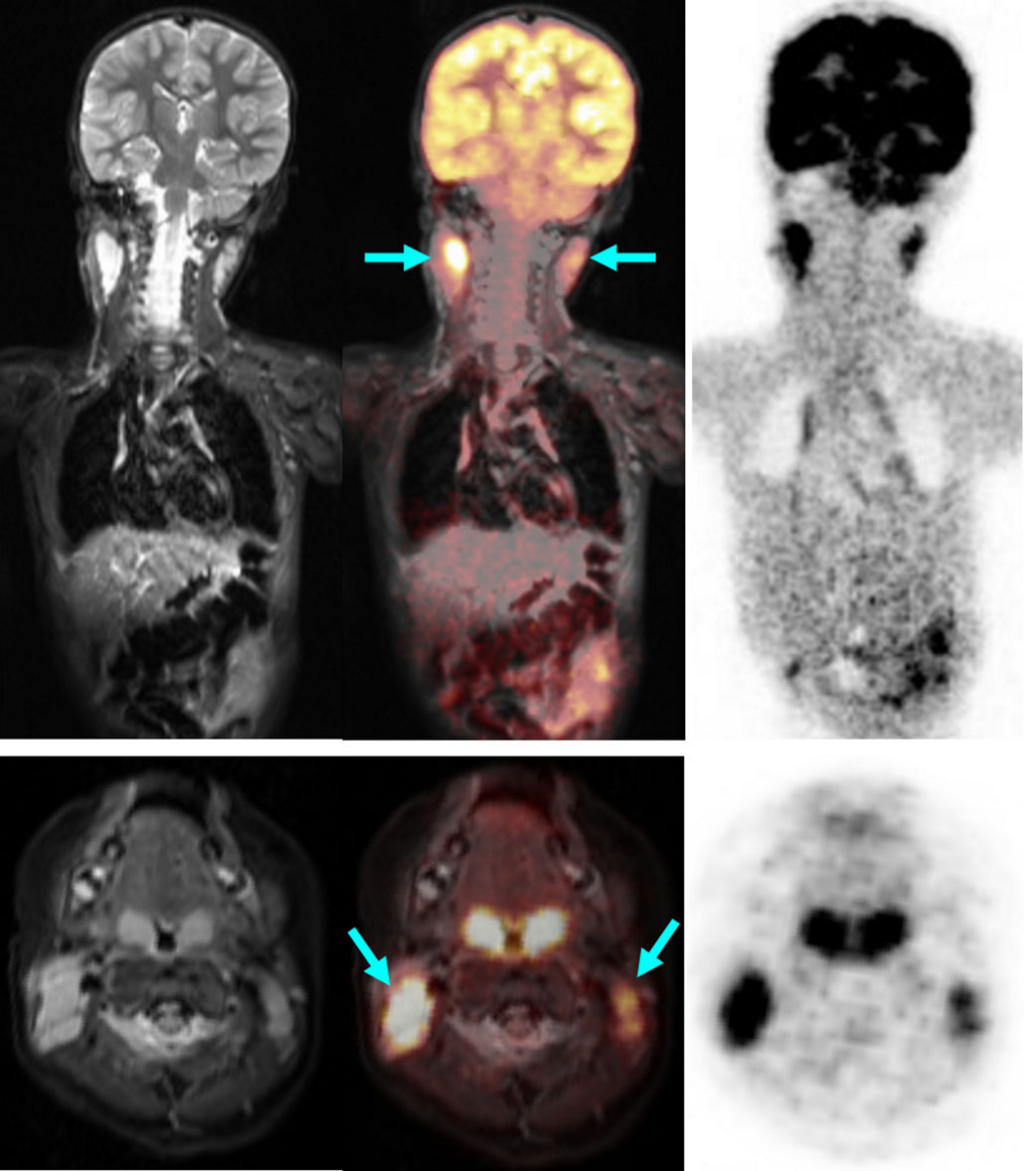
A 2.5-year-old boy with bilateral cervical (*arrows*) Burkitt lymphoma. *Arrows* indicate positive PET/MR findings

**Fig. 4:**
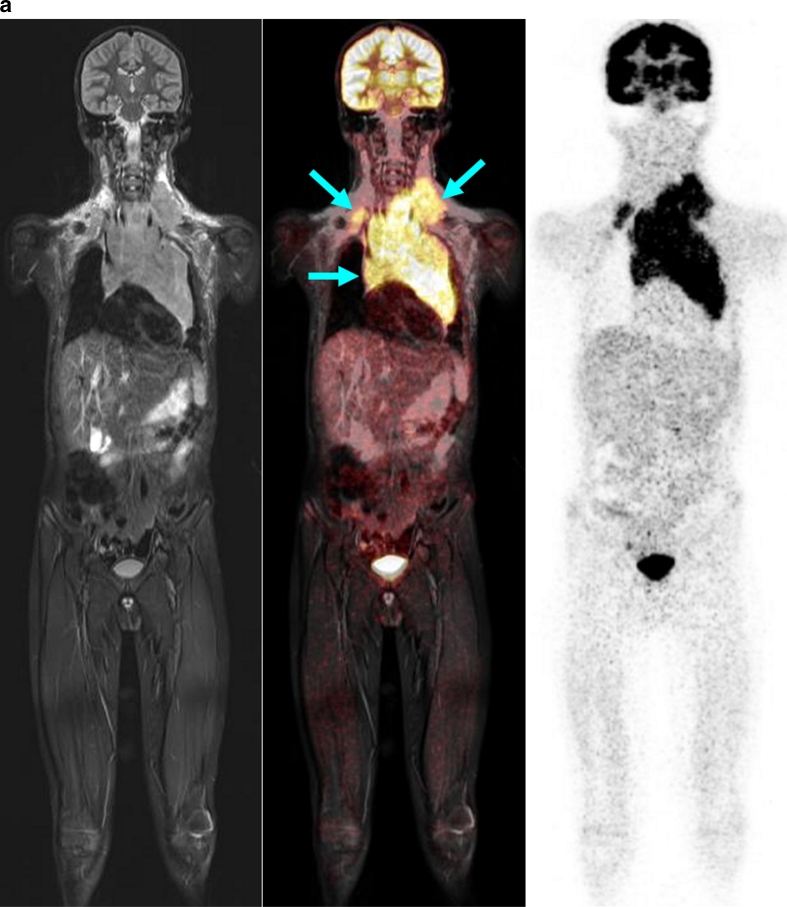

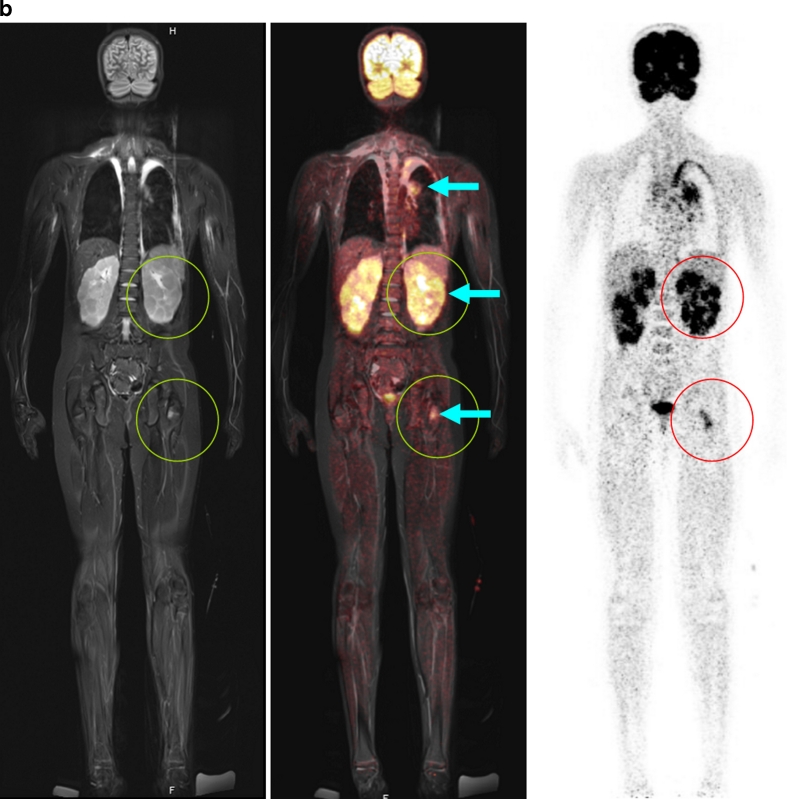

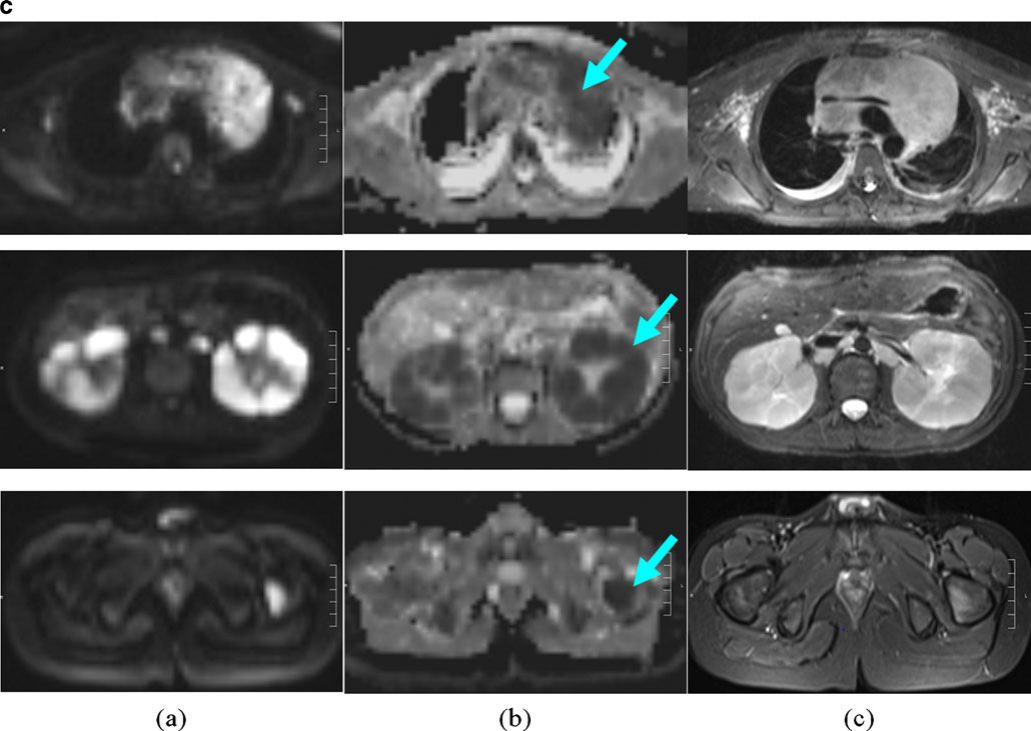
An 11-year-old boy with T-cell lymphoma with involvement of the neck, mediastinum, kidneys and bone marrow. **a** Significantly increased glucose metabolism of the pleural effusion also indicates tumour involvement in this region, while on MR this effusion was interpreted as an expression of congestion due to the large mediastinal tumour mass (*arrows*). **b** Note the significant pathological structure, size and metabolic activity (encircled, *arrows*) of the kidneys and the bone marrow in the left proximal femur. **c** The additional findings shown by the whole-body diffusion-weighted image (left column) and ADC map (middle column) show, concordant with PET findings, a distinct diffusion disorder in the area of the mediastinal mass (*top row*, *arrow*), the left kidney (*middle row*, *arrow*), and the proximal left femur (*bottom row*, *arrow*). Because of the bilateral similarity of the changes in the kidney, the interpretation of the PET scan was less certain. On the basis of significantly restricted diffusion and scale, the MR scan with diffusion-weighted image provided more conclusive identification of an active tumour tissue. *Arrows* in Fig. 4a and 4b indicate positive PET/MR findings. *Arrows* in Fig. 4c indicate positive ADC value typical for tumour tissue

Thus, the PET component proved to be appropriate and helpful for the detection of involved lymph nodes that were morphologically of borderline size but which on PET showed increased glucose metabolism, and for the characterization of slightly enlarged, but not involved, lymph nodes due to normal glucose metabolism (Figs. 2 and 3) [20]. In contrast, in the children studied, the MR examination demonstrated an advantage in those with symmetrical involvement of organs, as demonstrated by the example of a child with involvement of the testes (not shown) and the kidneys (Fig. 4). Because of the bilateral characteristics, the PET scan produced differential diagnostic uncertainties in the interpretation of these findings.

In addition, PET/MR provides options for whole-body diffusion imaging with B-values from 800 to 1,000, providing in individual cases an additional aid for differential diagnosis if the PET and MR findings produce differing interpretations [21]. Restricted diffusivity suggests increased cellularity and also a change in the nuclear–cytoplasmic ratio, and therefore suggests the possibility of metabolically active tumour tissue [22, 23].

### Solid child-like tumours

**Fig. 5:**
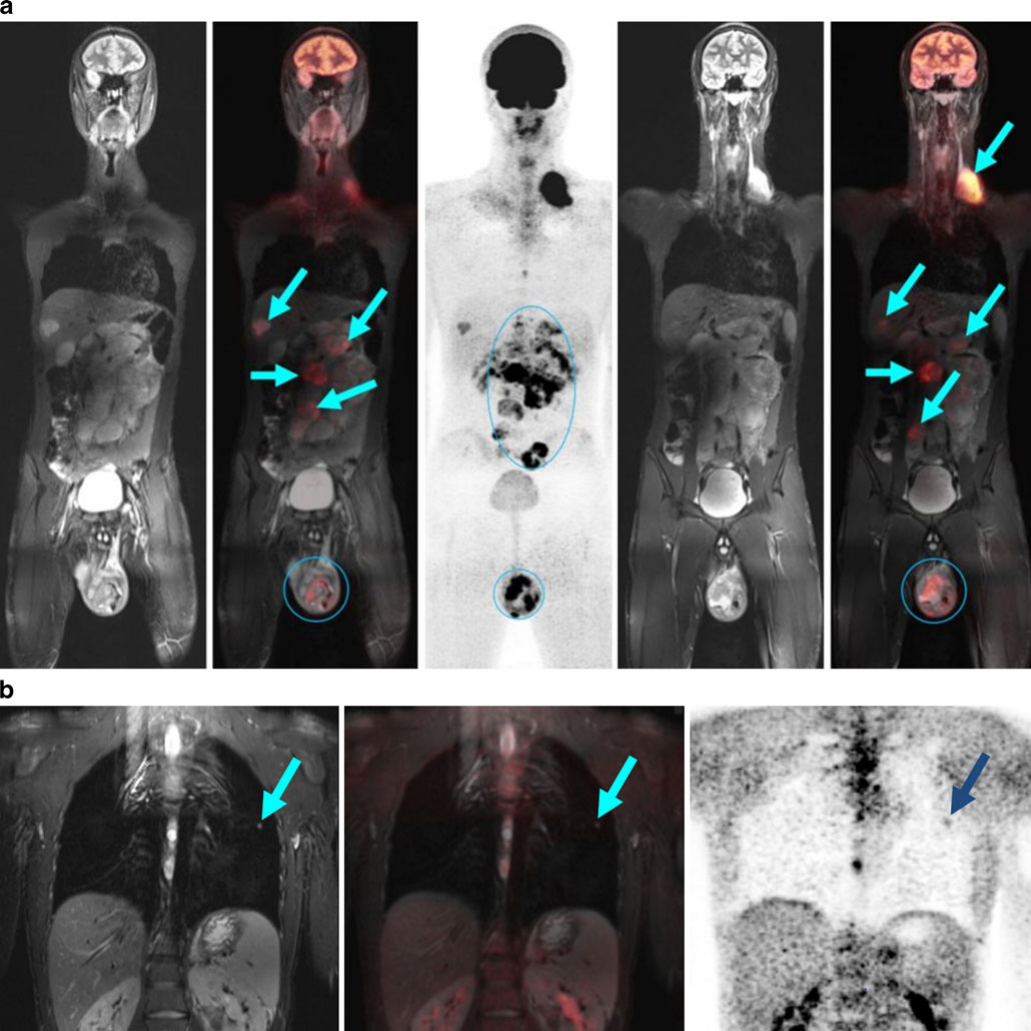
A 15-year-old boy with left testicular tumour with left retroperitoneal, supraclavicular and hepatic metastases (*arrows*). **a** The primary tumour in the left testicle and the retroperitoneal metastasis exhibit a very inhomogeneous enhancement of the glucose metabolism with larger metabolically inactive areas showing no enhancement. This can only be explained in part by tumour necrosis. The liver metastasis shows significantly enhanced glucose uptake. **b** A small metastasis (3 mm, *arrow*) in the left lung was diagnosed with good respiratory triggering by MR and by PET. This indicates that the system operates with high performance and yields superior images even of very small lesions. *Arrows* indicate positive PET/MR findings

**Fig. 6:**
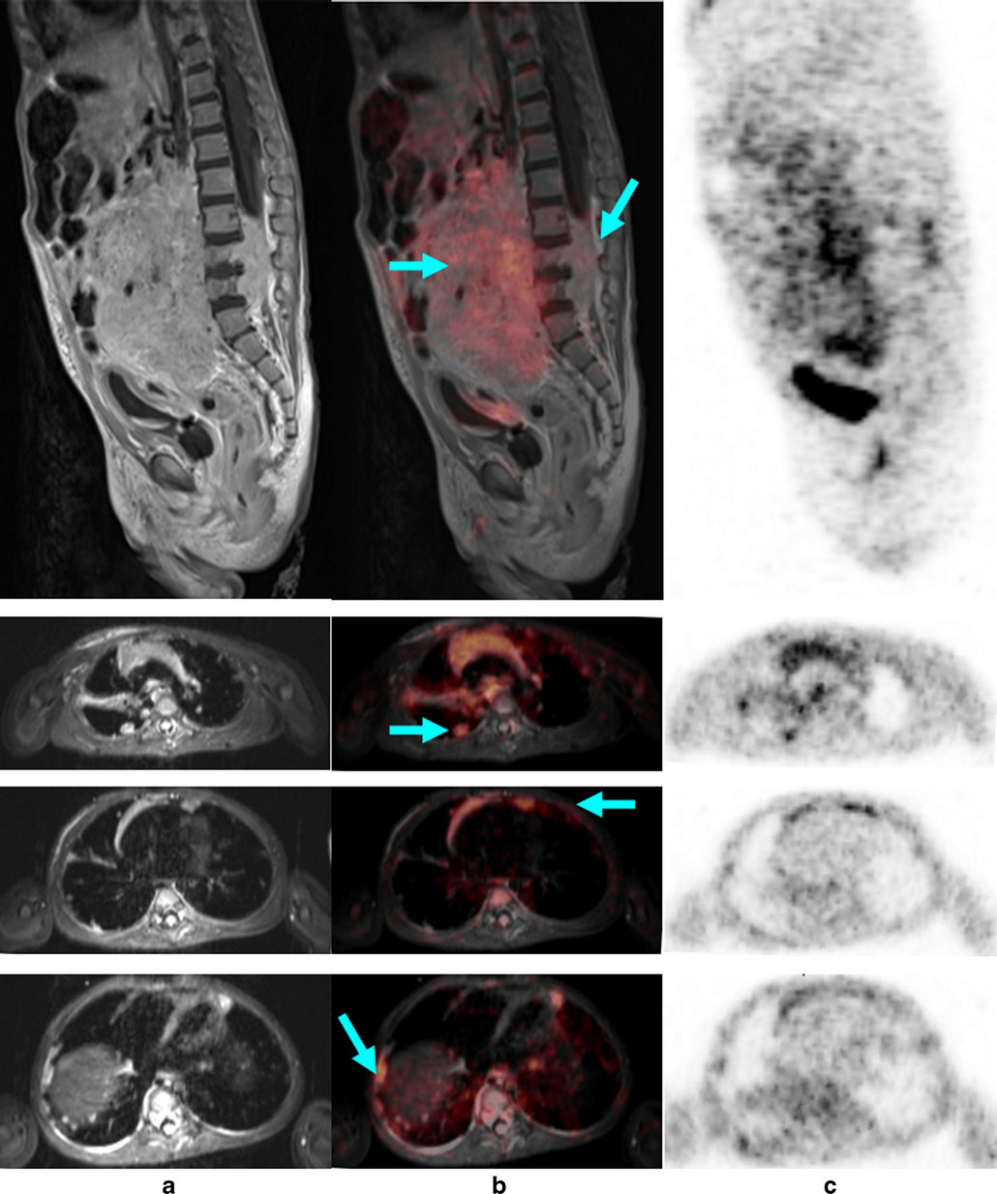
A 2-year-old boy with a large abdominal neuroblastoma that emanates from the sympathetic chain. *Top row*: the tumour (*arrows*) is growing into the spinal canal and involves the fourth and fifth lumbar vertebrae. The highly differentiated tumour shows only slightly intense inhomogeneous FDG uptake. *Lower row*: transaxial slices show multiple (*arrows*) lung metastases and pleural metastases. The metastases are seen on the concordant respiratory-triggered T2-weighted TIRM images (**a**) and on the PET images (**b, c**); smaller metastases of 3–5 mm are clearly shown on the MR images. *Arrows* indicate positive PET/MR findings

Even in solid tumours, the establishment of the metastasis and lymph node status using PET/MR is faster and clearer than with an isolated whole-body MR. In contrast, local staging, because of the spatial resolution required, is still the domain of dedicated MR imaging. Using the sequence protocols described above, we were able to reach a compromise, which in particular offered sufficient anatomical orientation for the paediatric surgeon, but nevertheless still embraced the concept of a one-stop-shop examination. The visually impressive fusion of high-resolution anatomy and metabolic activity was very positively rated by clinical colleagues. Because big tumours often exhibit large variability in local glucose uptake, the synergy of the two methods is obvious and can be used, for example, for the determination of the biopsy site and for treatment planning (Figs. 5 and 6).

FDG uptake may vary especially in neuroblastoma, and may diverge from more specific metaiodobenzylguanidine (MIBG) uptake labelling chromaffin tissues [24]. Since the intensity of FDG uptake in neuroblastoma is an adverse prognostic marker, FDG PET should be used in addition to MR and MIBG scintigraphy for the initial evaluation of high-risk neuroblastomas [25]. Small metastases (e.g. in the liver and lungs) usually show a concordant finding. Thus, in a child with a pulmonary metastasis that was only 3 mm in size, both methods were concordant, allowing a confident identification (Fig. 5). However, in the immediate future, lung CT will keep its place in the initial stages as a necessary supplementary modality during the transitional and evaluation phases. In most paediatric oncology protocols, lung CT scans are mandatory; only clear positive evidence from PET/MR will lead to a protocol change.

### Evaluation of treatment response and follow-up

**Fig. 7:**
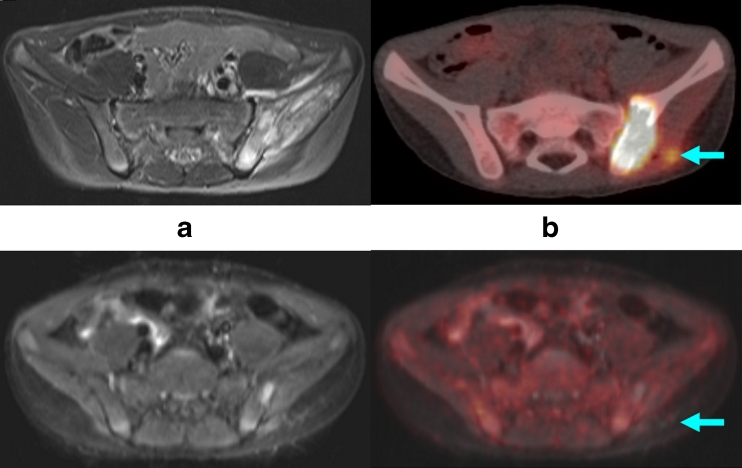
Anaplastic lymphoma with involvement of the ilium. *Top row*: initial, decisive MR image (**a**) and PET/CT image (*arrow*) (**b**). *Bottom row*: after treatment. In the whole-body MRI, residual bone lesions (*arrows*) can still be seen (**a**), which are not positive on the PET image (*arrow*) (**b**), and thus would be considered as inactive residual tissue

**Fig. 8:**
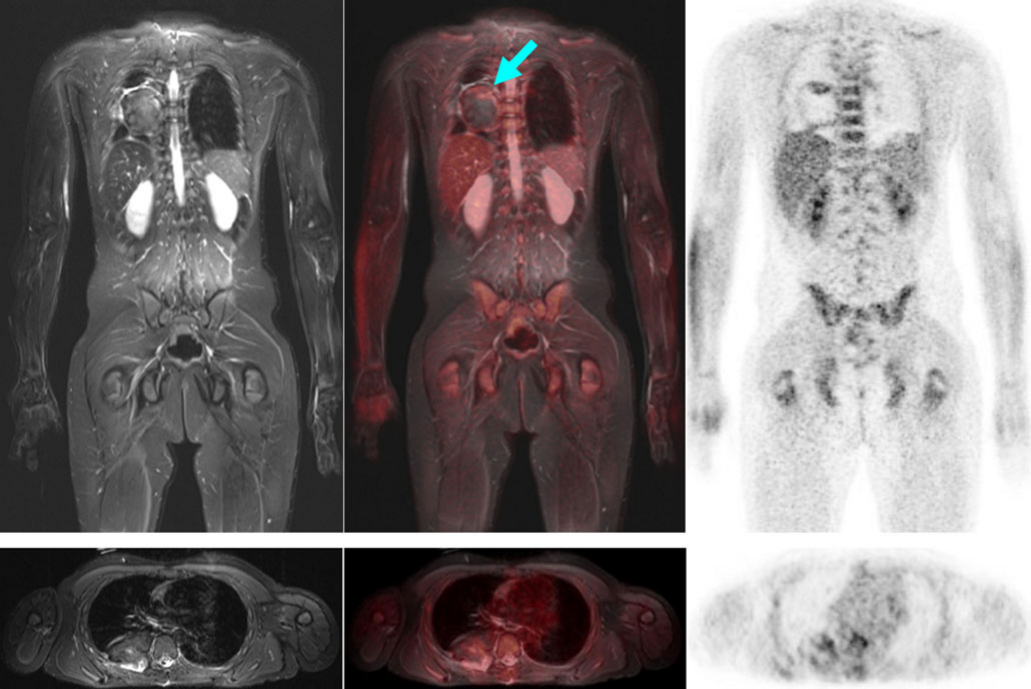
Large right-sided thoracic primitive neuroectodermal tumour residuum (*arrow*) in a 13-year-old boy at the end of neoadjuvant chemotherapy. The dark tumour on the T2-weighted MR image and mostly unenhanced FDG uptake on the PET image argue for predominant fibrous components. This was confirmed later histologically (Salzer-Kuntschik grade 2). However, in the cranial portion of the tumour there is a narrow zone of increased FDG uptake. On MRI, the still active residual tumour tissue was only ambiguously identified. *Arrow* indicates positive PET/MR finding

**Fig. 9:**
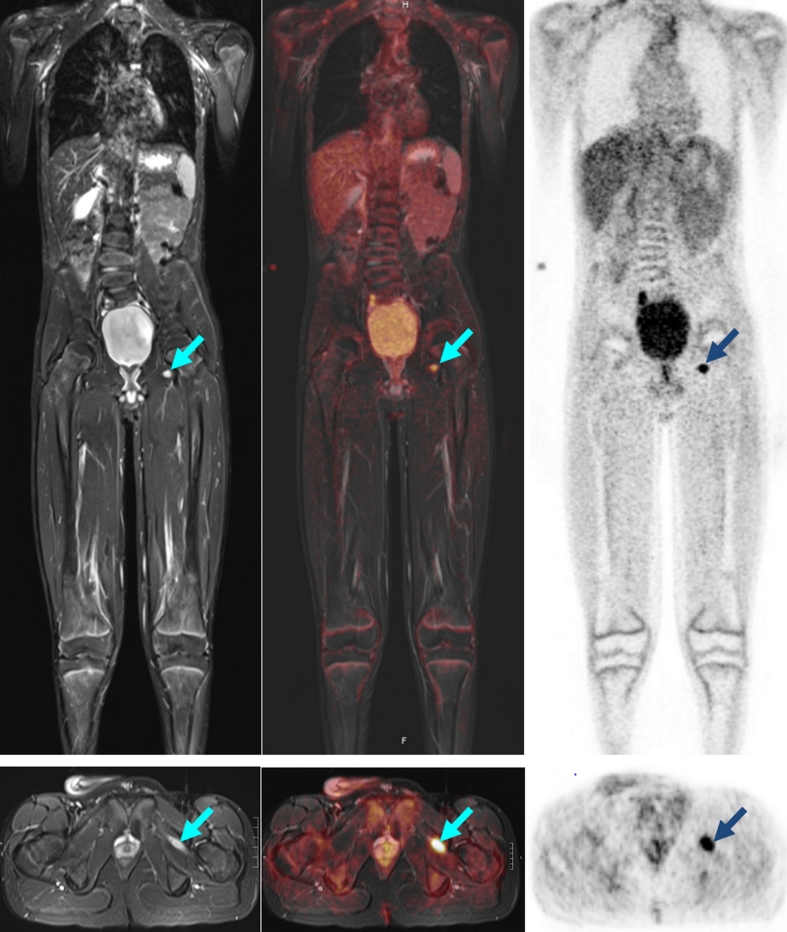
A 13-year-old boy with a history of extraosseous metastatic Ewing sarcoma of the pelvis now showing new imaging findings (*arrows*) suspicious for soft tissue metastasis in the adductor longus muscle. On MR imaging, size regression was seen within 7 weeks. Because of an intensive increase in glucose utilization, a biopsy was performed, which revealed inflammatory changes. *Arrows* indicate the positive PET/MR finding

Because of the novelty of PET/MR, we can at present only report follow-up studies in children in whom the initial PET/CT and MR investigations were performed consecutively. As shown in Fig. 7, lymphoma involvement of the ilium, initially demonstrated by PET/CT, was shown by PET to have normalized following chemotherapy, demonstrating an adequate treatment response. In contrast, MR imaging showed that a residual tumour mass was still present. In a child with thoracic primitive neuroectodermal tumour, PET during therapy showed that metabolically active tumour tissue remained in the periphery, while no reliable statements could be made following MR (Fig. 8). In a child with a history of metastatic extraosseous Ewing sarcoma, isolated PET findings indicated tumour recurrence, while the MR findings, on the basis of morphology and in comparison to a previous MR scan, indicated an inflammatory process (Fig. 9). The latter was confirmed histologically.

These examples illustrate that, as a rule, following chemotherapy, the metabolic reaction generally occurs sooner and more completely than the morphological reaction [26]. Therefore, for these therapeutic indications, PET is superior to MR alone. For the evaluation and localization of PET-positive residual findings, however, exact delineation of the anatomy at the same time is of great value. Regarding this issue, the complementary information given by PET/MR will be of increasing importance in the future [27].

### Limitations and pitfalls in PET/MR

In spite of the good overall image quality of both the MR and the PET components achieved with the acquisition protocol presented and the high technical success rate, several limitations have to be addressed. The typical limitations of whole-body MR persist even with PET/MR; for example, signal inhomogeneity was sometimes found in overlapping regions of the MR sections. Another critical factor was the calculation of the μ-map by means of the Dixon sequence [9]. Here we found inaccurate water/fat allocation in certain bed positions in five cases, which could have led to incorrect attenuation correction of the PET data. Whether this reduced the diagnostic value of the PET data in comparison to the PET/CT data cannot be assessed at this stage.

It is not yet possible to characterize the diagnostic accuracy of PET/MR. The aim of this paper is to share our first experience and to propose the first acquisition protocol for use in children. The scientific evaluation of PET/MR must now be the subject of prospective studies preferably with a multicentre design.

Another limitation of PET/MR is the huge amount of data produced by the Dixon sequence, the whole-body MR images and the PET data (approximately 2,800 images in total). Therefore, it is not possible to demonstrate a PET/MR study on a normal PACS workstation, even if the composite series only are transferred onto the PACS. The current version of syngo.via for PET/MR (Siemens, Erlangen, Germany) does not solve these problems sufficiently. Image transfer to the referring physician using a web application is no longer possible with the current web-based viewer solutions. So improved software solutions are required.

The main limitation of PET/MR, however, is the duration of the whole-body MR protocol. Due to respiratory triggering in the imaging of the thorax and upper abdomen, an ordinary MR acquisition lasts 45 min. Additional sequences for dedicated local diagnosis require a further 20–30 min. Normally, we allow the patient a period of rest away from the unit between these two parts of the study. Children under 6 years of age need anaesthesiological support.

## Conclusion

In children, minimizing the radiation dose is a consideration that determines the choice of imaging modality. With the introduction of PET/MR, the CT exposure component previously accrued in PET/CT studies can be eliminated [28]. In this way, the radiation exposure from a single hybrid imaging scan is reduced by around 80%, to only one effective dose of 4.6 mSv (two yearly doses of natural radiation exposure) [13].

The clinical advantages of PET/MR in children with oncological diseases above all rest in the significantly greater value that the MR component offers compared to conventional CT. MR is capable of identifying the anatomy, perfusion and diffusion of a tumour [29]. In addition, the soft-tissue resolution of MR is clearly superior to that of CT [30]. The simultaneously measured PET signal provides the established advantages of function determination and quantification by means of SUV [31–33].

Our initial experience in children suffering from multifocal, oncological diseases shows that a complete PET/MR examination that is completed within 60 min provides all relevant information for paediatric surgeons and paediatric oncologists. As a result, the reduction in time required for the diagnostic procedure in the initial stages is relevant. All of these established advantages show that in children with oncological diseases combined PET/MR including whole-body diffusion weighting demonstrates a new quality in imaging and functional diagnostics [34].

## References

[1] Wehrl HF, Judenhofer MS, Wiehr S (2009). Pre-clinical PET/MR: technological advances and new perspectives in biomedical research. Eur J Nucl Med Mol Imaging.

[2] Sauter AW, Wehrl HF, Kolb A (2010). Combined PET/MR: one step further in multimodality imaging. Trends Mol Med.

[3] Hall EJ, Brenner DJ (2008). Cancer risks from diagnostic radiology. Br J Radiol.

[4] Brenner DJ, Sachs RK (2006). Estimating radiation-induced cancer risks at very low doses: rationale for using a linear no-threshold approach. Radiat Environ Biophys.

[5] Schlemmer HP, Pichler BJ, Krieg R (2009). An integrated MR/PET system: prospective applications. Abdom Imaging.

[6] von Schulthess GK, Burger C (2010). Integrating imaging modalities: what makes sense from a workflow perspective?. Eur J Nucl Med Mol Imaging.

[7] Biograph mMR. Datasheets. Siemens. www.siemens.com/mMR. Accessed 9 Nov 2012

[8] Delso G, Fürst S, Jakoby B (2011). Performance measurements of the Siemens mMR integrated whole-body PET/MR scanner. J Nucl Med.

[9] Eiber M, Martinez-Möller A, Souvatzoglou M (2011). Value of a Dixon-based MR/PET attenuation correction sequence for the localization and evaluation of PET-positive lesions. Eur J Nucl Med Mol Imaging.

[10] Hofmann M, Bezrukov I, Mantlik F (2011). MR-based attenuation correction for whole-body PET/MR: quantitative evaluation of segmentation- and atlas-based methods. J Nucl Med.

[11] Stauss J, Franzius C, Pfluger T, European Association of Nuclear Medicine (2008). Guidelines for 18F-FDG PET and PET-CT imaging in paediatric oncology. Eur J Nucl Med Mol Imaging.

[12] Fahey FH, Treves ST, Adelstein SJ (2011). Minimizing and communicating radiation risk in pediatric nuclear medicine. J Nucl Med.

[13] Chawla SC, Federman N, Zhang D (2010). Estimated cumulative radiation dose from PET/CT in children with malignancies: a 5-year retrospective review. Pediatr Radiol.

[14] Krohmer S, Sorge I, Krausse A (2010). Whole-body MR for primary evaluation of malignant disease in children. Eur J Radiol.

[15] Schwenzer NF, Schmidt H, Claussen CD (2012). Whole-body MR/PET: applications in abdominal imaging. Abdom Imaging.

[16] Antoch G, Vogt FM, Freudenberg LS (2003). Whole-body dual-modality PET/CT and whole-body MR for tumor staging in oncology. JAMA.

[17] Tsushima Y, Takano A, Taketomi-Takahashi A (2007). Body diffusion-weighted MR imaging using high b-value for malignant tumor screening: usefulness and necessity of referring to T2-weighted images and creating fusion images. Acad Radiol.

[18] Hirsch W, Sorge I, Krohmer S (2008). MR of the lungs in children. Eur J Radiol.

[19] Franzius C, Stauss J, Pfluger T (2010). Procedure guidelines for whole-body 18F-FDG PET and PET/CT in children with malignant diseases. Nuklearmedizin.

[20] Kluge R, Körholz D (2011). Die Bedeutung des FDG-PET für die Stadieneinteilung und Therapie des Hodgkin-Lymphoms im Kindesalter [Role of FDG-PET in staging and therapy of children with Hodgkin lymphoma]. Klin Padiatr.

[21] Takenaka D, Ohno Y, Matsumoto K (2009). Detection of bone metastases in non-small cell lung cancer patients: comparison of whole-body diffusion-weighted imaging (DWI), whole-body MR imaging without and with DWI, whole-body FDG-PET/CT, and bone scintigraphy. J Magn Reson Imaging.

[22] Takahara T, Imai Y, Yamashita T (2004). Diffusion weighted whole body imaging with background body signal suppression (DWIBS): technical improvement using free breathing, STIR and high resolution 3D display. Radiat Med.

[23] King AD, Ahuja AT, Yeung DK (2007). Malignant cervical lymphadenopathy: diagnostic accuracy of diffusion-weighted MR imaging. Radiology.

[24] Melzer HI, Coppenrath E, Schmid I (2011). 123I-MIBG scintigraphy/SPECT versus 18F-FDG PET in paediatric neuroblastoma. Eur J Nucl Med Mol Imaging.

[25] Papathanasiou ND, Gaze MN, Sullivan K (2011). 18F-FDG PET/CT and 123I-metaiodobenzylguanidine imaging in high-risk neuroblastoma: diagnostic comparison and survival analysis. J Nucl Med.

[26] Cheson BD, Pfistner B, Juweid ME (2007). International harmonization project on lymphoma. Revised response criteria for malignant lymphoma. J Clin Oncol.

[27] Histed SN, Lindenberg ML, Mena E (2012). Review of functional/anatomical imaging in oncology. Nucl Med Commun.

[28] Pichler BJ, Kolb A, Nägele T (2010). PET/MR: paving the way for the next generation of clinical multimodality imaging applications. J Nucl Med.

[29] Chavhan GB, Babyn PS (2011). Whole-body MR imaging in children: principles, technique, current applications, and future directions. Radiographics.

[30] Goo HW, Choi SH, Ghim T (2005). Whole-body MR of paediatric malignant tumours: comparison with conventional oncological imaging methods. Pediatr Radiol.

[31] Jadvar H, Connolly LP, Fahey FH (2007). PET and PET/CT in pediatric oncology. Semin Nucl Med.

[32] Weckesser M (2009). Molecular imaging with positron emission tomography in paediatric oncology – FDG and beyond. Pediatr Radiol.

[33] Purz S, Mauz-Körholz C, Körholz D (2011). [18F]Fluorodeoxyglucose positron emission tomography for detection of bone marrow involvement in children and adolescents with Hodgkin’s lymphoma. J Clin Oncol.

[34] Kwee TC, Takahara T, Vermoolen MA (2010). Whole-body diffusion-weighted imaging for staging malignant lymphoma in children. Pediatr Radiol.

